# Association of visual impairment with malnutrition among elderly Indians: A pilot study

**DOI:** 10.6026/9732063002002024

**Published:** 2024-12-31

**Authors:** Rahul Kumar Verma, Shrinkhal, Koushik Biswas

**Affiliations:** 1 All India Institute of Medical Sciences, Raebareli, Uttar Pradesh, India; 2 Department of Ophthalmology, All India Institute of Medical Sciences, Raebareli, Uttar Pradesh, India; 3 Department of Biochemistry, All India Institute of Medical Sciences, Raebareli, Uttar Pradesh, India

**Keywords:** Visual impairment, aged, geriatric assessment, nutrition assessment, malnutrition, mini nutritional assessments, dietary patterns

## Abstract

The elderly with visual impairment in India are at increased risk of malnutrition. Hence, a cross-sectional study of 50 visually impaired (visual acuity <6/18) and 50 normal vision elderly participants by convenience sampling at the ophthalmology clinic was completed. Interview and nutritional assessment of participants using Mini Nutritional Assessment were carried out. Visually impaired elderly reported less frequent consumption of dairy products (52% vs. 58%), legumes or eggs (82% vs. 94%) and fruits or vegetables (80% vs. 90%), compared to those with normal vision. Logistic regression analysis revealed visual impairment as an independent risk factor for malnutrition in the elderly (OR 8.41, 95% CI 1.52 - 46.36, p=0.015).

## Background:

India is presently in the stage three of demographic transition, where a low birth rate, low fertility and decline in death rate is leading to an increase in the proportion of elderly population [1]. As per 2021 reports, India has over 138 million elderly people (60 years and above) who comprise 10.1% of the national population [2]. The physiological functions decrease with increasing age. The highest prevalence of disability and functional impairment is reported in people belonging to the age group 60 years and above [3]. Sensory problems such as visual impairment are common among elderly people and their prevalence increases with increasing age [4, 5]. Visual impairment restricts mobility and social contact and is known to be a risk factor for disability. Elderly people with visual impairment are reported to face three times more difficulty in managing medication and 3.5 times more difficulty in preparing a meal compared to those who do not have a visual problem [6]. People with visual impairment are reported to have numerous barriers to health information and are more likely to be below the poverty threshold [5, 7]. Although elderly people with visual impairment may be at greater risk of poor nutritional status due to a loss in functional capacity, difficulty in shopping, cooking and eating meals, few studies have explored these associations among elderly people [4]. Payette *et al.* [8] reported that an independent association exists between poor vision and lower energy and protein intake. A study conducted in the United States of America reported that the prevalence of underweight is higher among elderly suffering from blindness [9]. Malnutrition generally occurs when there is an imbalance between the nutrient intake and the body's requirements over a prolonged period. This affects the body weight, composition and physical functions over the time course [10]. There is no single "gold standard" method for the assessment of nutritional status in the elderly [11]. This is mainly because there are multiple causes of malnutrition in the elderly. Biochemical markers, clinical indices, anthropometry and functional capacity have been taken into consideration while developing nutritional screening tools for early detection of malnutrition in the elderly. Some of these tools include the Geriatric Nutritional Risk Index (GNRI), Nutritional Risk Index (NRI), Subjective Global Assessment (SGA), Nutrition Risk Screening, Mini Nutritional Assessment (MNA) and "Malnutrition Universal Screening Tool" (MUST) [12, 13]. Guigoz and Vellas reported that Mini Nutritional Assessment (MNA) has 96 % sensitivity, 98% specificity and 97 % predictive value [14]. Pascolini and Mariotti [15] reported that the burden of visual impairment in India is about 62 million, out of which 54 million have low vision and 8 million are blind. Due to an increase in life expectancy, the number of elderly people in India is continuously rising [16]. Therefore, it is of interest to evaluate if the elderly with visual impairment in India are at greater risk of malnutrition.

## Materials and Methods:

## Study setting:

The study subjects were recruited and data were collected in the Ophthalmology Outpatient Department (OPD) of All India Institute of Medical Sciences, Raebareli a tertiary care teaching hospital.

## Study design:

This is a cross-sectional study.

## Study population:

## Inclusion criteria:

Elderly people aged 65 years and above attending the Ophthalmology OPD.

## Exclusion criteria:

Those on a diet or exercise regime for weight loss

Those with a coexisting malignancy or a history of malignancy

Those who are unable to give consent for the study due to any psychiatric illness or other health condition.

## Sample size:

One hundred elderly subjects (50 with normal vision and 50 with impaired vision) were selected for our study by convenience sampling. In the ophthalmology OPD, the first 50 elderly attending with normal vision were categorized under the "normal vision" group, while the first 50 elderly attending with impaired vision were categorized under the "visually impaired" group.

## Study duration:

Data was collected from November to December 2023 and analyzed over the next two months.

## Data collection:

Visual impairment was defined as visual acuity of worse than 6/18 in the better eye with available correction [17]. The distant visual acuity of literate participants was tested by Snellen's test types, while for illiterate participants, Landolt's C chart or E chart was used. Based on the visual acuity, the participants were categorized as "normal vision" or "visually impaired". A case report form (CRF) and Mini Nutritional Assessment (MNA) were filled for all participants. The CRF was pre-designed and pre-validated before use. The CRF was used to gather information from the participants. Information collected included participant's name, age, sex, hospital registration number, address, presenting complaint, provisional diagnosis, co-existing illness, visual acuity, addiction history, MNA screening score and malnutrition indicator score. The medical history was obtained from patient records. The MNA tool (developed by the Nestle Nutrition Institute) consisted of simple measurements and brief questions which could be completed in 15 minutes for each participant. The questions included anthropometric measurement (height, weight, mid-arm circumference, calf circumference), global assessment (questions related to lifestyle, medication, mobility), dietary questionnaire (questions related to the number of meals, food, fruit, protein and fluid intake, the autonomy of feeding) and subjective assessment (self-perception of health and nutrition). The maximum score of the MNA was 30, with a higher score indicating better nutritional status. A score of less than 17 indicated malnutrition, between 17 and 23.5 indicated at risk of malnutrition and a score from 24 to 30 indicated normal nutritional status. A pre-validated Mini Nutritional Assessment (MNA) in the local language (Hindi) was used for patient convenience. The first author was trained by the third author on the art of asking questions from the study participants in the local language (Hindi) and taking anthropometric measurements for a week. Thereafter, the first author collected the participant's data under supervision. Height was measured by a portable stadiometer SECA 213 and weight by a flat digital scale SECA 813. Mid-arm circumference and calf circumference were measured with an ergonomic circumference measuring tape SECA 201.

## Ethical considerations:

Ethical clearance was taken from the Institutional Ethics Committee before starting the study (Approval number: 2023 -20 - EMP (STS) - 5 dated 09.11.2023). A written informed consent was taken from all the study participants prior to their enrolment into the study. Confidentiality of participant data was maintained at all levels.

## Statistical analysis:

All participant data were entered in a Microsoft Excel spreadsheet. The categorical data were presented as frequency and percentage. Pearson's chi-square test was used to identify any statistically significant differences between the categorical data. The continuous data was checked for normality using the Kolmogorov-Smirnov test. All parametric data were presented as mean ± standard deviations, while the non-parametric data were presented as median and interquartile range. An independent sample t-test was used to calculate the significance of study parameters between two unrelated groups for parametric data. A logistic regression test was used to determine if visual impairment increased the chances of malnutrition. A p-value of <0.05 was considered as a level of significance. Data was analyzed using IBM SPSS version 25.

## Results:

Our study included 100 participants of which 50 had normal vision and 50 had impaired vision. The median age of participants was 66 years (range, 65 - 83 years). The majority (65 %) of the participants were in the 65-69 years age group. Out of the 100 participants, 57 (57 %) were male. The age and sex distribution of the participants is mentioned in Table 1. On analyzing the past medical history and associated comorbidities of participants, we observed that the prevalence of coexisting hypertension, diabetes mellitus, chronic kidney disease, chronic liver disease, history of cerebrovascular accident and history of hip fracture was higher among the visually impaired, however, this was not statistically significant. Substance abuse was also more common among the visually impaired; however, this finding was statistically insignificant (Table 2).

Next, we analyzed the dietary habits of the participants from the MNA form. We observed that a higher number of participant with visual impairment reported a decrease in food intake over the last three months (34 % vs. 28 %), less opportunity to eat three full meals daily (14 % vs. 20 %), lower consumption of dairy products daily (52 % vs. 58 %), lower consumption of legumes or eggs weekly (82 % vs. 94 %), lower intake of meat, fish or poultry regularly (2 % vs. 4 %) and a lower consumption of fruits or vegetables serving daily (80 % vs. 90 %), compared to participants with normal vision, however, these were not statistically significant. The consumption of water and fluids was significantly less in visually impaired participants compared to those with normal vision (34 % vs. 70 %, χ^2^=14.33, p = 0.001). The ability to self-feed was significantly lower among participants with impaired vision compared (90% vs. 100 %, χ^2^=5.26, p = 0.022) (Table 3). 4A significantly higher number visual impaired participants had a self-view of suffering from a nutritional problem compared to those with normal vision (34 % vs. 12 %, χ^2^=7.75, p = 0.021). Analyzing the anthropometric indices revealed no significant statistical difference in the body mass index of participants in both the groups. However, the visually impaired had a significantly lower mid-arm circumference (24.94 ± 3.17 vs. 26.73 ± 3.35, t = -2.75, p = 0.007) and calf circumference (30.16 ± 3.40 vs. 32.11 ± 3.19, t = -2.96, p = 0.004) (Table 4). Based on the malnutrition indicator score we observed that among the visually impaired, 9 (18 %) were malnourished, 28 (56 %) were at risk of malnutrition and 13 (26 %) had a normal nutritional status. Among those with normal vision, we observed that 2 (4 %) were malnourished, 20 (40 %) were at risk of malnutrition and 28 (56 %) had a normal nutritional status (Figure 1 - see PDF). In logistic regression analysis, adjusting for age, sex, hypertension and diabetes mellitus, visual impairment was an independent predictor of malnutrition (MNA score < 17) in the elderly (OR 8.41, 95% CI 1.52 - 46.36, p=0.015).

## Discussion:

India is presently witnessing a decline in birth rate and an increase in life expectancy which is leading it towards a "greying" population [18]. By the year 2050, the elderly will comprise 20 % of India's population [19]. An important aspect of the elderly is a gradual decline of physiological functions. Optimum nutrition and regular physical activity are important to preserve function and well-being [20]. Socio-economic problems, chewing problems, swallowing problems, living alone or in a nursing home, loss of mobility and various acute and chronic diseases are reported to be risk factors for anorexia, which predisposes the elderly to malnutrition and has a negative health outcome [21]. A previous study conducted in Finland reported visual impairment as an independent predictor of malnutrition in the elderly population residing in assisted living facilities [4]. However, the findings of this study cannot be extrapolated in the Indian context, where the majority of the elderly reside with their children and grandchildren, which are known to have a positive effect on their health outcomes [22]. In this study, we identified, that the elderly in India with visual impairment have 8.41 times higher chances of suffering from malnutrition (95% CI 1.52- 46.36, p=0.015). In this study, we identified that among the visually impaired elderly participants, 18 % were malnourished and 56 % were at risk. In contrast, among the visually impaired elderly in Finland, 26.1 % were malnourished and 57.6 % were at risk [4]. We identified that mid-arm circumference and calf circumference may be better predictors of malnutrition in the elderly compared to body mass index. Tsai *et al.* [23] also reported that mid-arm circumference and calf circumference were more effective than body mass index in predicting mortality risk for elderly patients in Taiwan. Payette *et al.* [8] reported that the majority of the functionally dependent elderly population in Canada did not consume recommended levels of dietary proteins and identified poor appetite, disease burden, stress and poor vision as independent predictors of low protein intake. In this study, the significantly lower mid-arm circumference and calf circumference in elderly with visual impairment may be a result of inadequate protein intake over a long period. Although statistically insignificant, fewer visually impaired participants in our study consumed dairy products, legumes or eggs regularly compared to those with normal vision (Table 3). However, regular consumption of dairy products was identified in the majority (over 50%) of participants (Table 3). This may be because a significant proportion of the people living in the Indian state of Uttar Pradesh consume a lacto-vegetarian diet due to cultural and religious issues [24]. Most government-sponsored nutritional intervention programs in India are targeted to improve the health outcomes of children and reproductive women. Malnutrition in the elderly is often undiagnosed. Early identification and timely intervention of malnutrition in the elderly are likely to prevent adverse health outcomes [25]. In this study, we identified that among the elderly with normal vision, 4 % were malnourished and 40 % were at risk. Sullar *et al.* [26] reported that among the elderly residents of the tea gardens of the North Bengal region of India, 6.1 % were malnourished and 64.6 % were at risk. Lahiri *et al.* [27] reported that among the elderly residents of a rural area in eastern India, 29.4 % were malnourished and 60.4 % were at risk. Based on the findings of our study we recommend introducing nutritional screening for the elderly with visual impairment under the National Programme for Control of Blindness. We expect it can help in early identification of malnutrition in elderly and prompt intervention. Our study was not without limitations. The study was conducted on patients attending the hospital, who may not always be representative of the population in the community. The sample size in our study was less. As this is a student project that had to be completed within a time frame of two months, we limited our sample size to 100 elderly participants. We used only the MNA tool to assess malnutrition and did not use any other tool or biochemical marker for our study. Further research can be conducted in this area with a larger sample size in the community setting.

## Conclusion:

Our findings highlight that visual impairment is an independent predictor of malnutrition among the elderly population of India. Introducing nutritional screening for the elderly with visual impairment under the National Programme for Control of Blindness can help in early identification of malnutrition and prompt intervention. In the resource-constrained rural Indian setting, the Ophthalmologist working in the Community Health Centre or District Hospital can train his team of health workers to screen the elderly for malnutrition using the Mini Nutritional Assessment or similar tools.

## Figures and Tables

**Table 1:** Age and sex distribution of participants

**Characteristic**		**Visually impaired n=50**	**Normal vision n=50**	**Total n=100**
Age (in years)	65 - 69	31 (62 %)	34 (68 %)	65 (65 %)
	70 - 74	6 (12 %)	13 (26 %)	19 (19 %)
	75 - 79	8 (16 %)	3 (6 %)	11 (11 %)
	≥ 80	5 (10 %)	0 (0 %)	5 (5 %)
Sex	Male (%)	23 (46 %)	34 (68 %)	57 (57 %)
	Female (%)	27 (54 %)	16 (32 %)	43 (43 %)

**Table 2:** Previous medical history and comorbidities of participants

**Characteristic**	**Visually impaired n=50**	**Normal vision n=50**	**Pearson's Chi-square**	**p-value**
Hypertension	24 (48 %)	22 (44 %)	0.16	0.688
Diabetes mellitus	14 (28 %)	13 (26 %)	0.05	0.822
Chronic kidney disease	3 (6 %)	2 (4 %)	-	-
Chronic liver disease	3 (6 %)	1 (2 %)	-	-
CVA	4 (8 %)	1 (2 %)	-	-
Fracture	3 (6 %)	2 (4 %)	-	-
Mobility	48 (96 %)	50 (100 %)	2.04	0.15
Addiction	9 (18 %)	3 (6 %)	3.41	0.065

**Table 3:** Dietary habits of study participants

**Characteristic**	**Visually impaired n=50**	**Normal vision n=50**	**Pearson's Chi-square test**	**p-value**
Food intake has decreased in the last 3 months	17 (34 %)	14 (28 %)	2.24	0.326
Takes 3 full meals daily	7 (14 %)	10 (20 %)	4.56	0.103
Takes at least one serving of dairy products daily	26 (52 %)	29 (58 %)	0.36	0.546
Takes two or more servings of legumes or eggs per week	41 (82 %)	47 (94 %)	3.41	0.065
Takes meat fish or poultry every day	1 (2 %)	2 (4 %)	-	-
Takes two or more servings of fruits or vegetables every day	40 (80 %)	45 (90 %)	1.96	0.161
Takes more than 5 cups of fluid daily	17 (34 %)	35 (70 %)	14.33	0.001
Can able to self-feed without any problem	45 (90 %)	50 (100 %)	5.26	0.022

**Table 4:** Anthropometric indices of study participants

**Characteristic**	**Visually impaired n=50**	**Normal vision n=50**	**Unpaired t-test**	**p-value**
Body Mass Index (in kg/m^2^)	23.33±6.88	24.99±4.11	-1.47	0.146
Mid arm circumference (in cm)	24.94±3.17	26.73±3.35	-2.75	0.007
Calf Circumference (in cm)	30.16±3.40	32.11±3.19	-2.96	0.004
